# Nanoconfined Crystallization for High‐Efficiency Inorganic Perovskite Solar Cells

**DOI:** 10.1002/smsc.202000054

**Published:** 2021-01-15

**Authors:** Xiao Jiang, Kai Wang, Hui Wang, Lianjie Duan, Minyong Du, Likun Wang, Yuexian Cao, Lu Liu, Shuping Pang, Shengzhong (Frank) Liu

**Affiliations:** ^1^ Dalian National Laboratory for Clean Energy iChEM Dalian Institute of Chemical Physics Chinese Academy of Sciences Dalian Liaoning 116023 China; ^2^ University of the Chinese Academy of Sciences Beijing 100039 China; ^3^ Qingdao Institute of Bioenergy and Bioprocess Technology Chinese Academy of Sciences Qingdao 266101 China; ^4^ Key Laboratory of Applied Surface and Colloid Chemistry Ministry of Education Shaanxi Key Laboratory for Advanced Energy Devices Shaanxi Engineering Lab for Advanced Energy Technology Institute for Advanced Energy Materials School of Materials Science and Engineering Shaanxi Normal University Xi'an 710119 China

**Keywords:** inorganic perovskites, mesoporous silica, nanoconfined crystallization, solar cells

## Abstract

Given that thermal stability is of considerable importance in the field of photovoltaics, inorganic perovskites have attracted numerous attempts to overcome instability caused by volatile cations in organic–inorganic hybrid perovskites. As always, crystallization optimization is a paramount strategy to enhance the performance of inorganic perovskite‐based solar cells. Recently, nanoconfined crystallization is regarded as a novel and effective strategy due to the absence of chemical reactions. Herein, 1D ordered mesoporous silica is introduced into inorganic perovskite precursors to facilely induce the nanoconfined crystallization. Both theoretical and experimental analyses verify that the nanoconfined crystallization is successfully triggered by the ordered mesoporous silica, fostering the formation of 1D perovskite monocrystal. In addition, the crystallization and morphology of inorganic perovskite are effectively facilitated. As a result, the nonradiative recombination is suppressed along with the distinctly reduced trap‐state density and remarkably enhanced charge transport in perovskite. Finally, the power conversion efficiencies of CsPbIBr_2_‐ and CsPbI_3_‐based solar cells are boosted from 8.67% to 10.04% and from 14.10% to 14.69%, respectively. Meanwhile, stability tests of solar cells also show enhancement using the nanoconfined crystallization. This work provides a facile, effective, and flexible crystallization modulating strategy for fabricating efficient and stable inorganic perovskite solar cells.

## Graphical abstract

ZrSBA‐15 is utilized in the nanoconfined crystallization of inorganic perovskites for the optimization of lattice strain as well as the enlargement of grain size. The as‐prepared perovskite solar cells thus obtain well‐promoted power conversion efficiency (PCE).

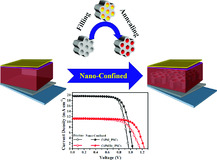

## 1 Introduction

Organometal hybrid halide perovskites have been regarded as preeminent light‐harvesting materials owing to their optoelectrical advantages, such as high carrier mobility, long carrier life time, and tunable optical adsorption.^[^ ^1^, ^2^, ^3^, ^4^, ^5^ ^]^ To date, perovskite solar cells (PSCs) have attained mushrooming power conversion efficiency (PCE) from an initial value of 3.8–25.2%.^[^ ^6^, ^7^, ^8^, ^9^ ^]^ Despite the outstanding PCE, organic–inorganic PSCs suffer from the thermal and photoinstabilities inherited from volatile organic components, which is a critical ﬂaw for further applications. Therefore, cesium cation (Cs^+^)‐based inorganic perovskites (CsPbX_3_, X = I or Br) have attracted immense interests in the field of photovoltaics on account of the nonvolatility.^[^ ^10^ ^]^

Among inorganic perovskites, CsPbI_3_ equips the narrowest bandgap (1.73 eV) and PSCs fabricated with it can achieve a PCE exceeding 19%,^[^ ^11^ ^]^ whereas it unfortunately possesses extremely toilless structure transition toward the inactive *δ*‐phase. Entirely or partially substituting iodide with bromide enables the phase stability enhancement at the cost of increased bandgap. Then, CsPbI_2_Br gradually came to the stage with a suitable bandgap (1.9 eV) and the corresponding PSCs have attained a champion PCE of 16.8%.^[^ ^12^ ^]^ Certainly, CsPbI_2_Br still sustains friable phase stability under moisture conditions. Given this, Br‐rich inorganic perovskites of CsPbIBr_2_ (2.05 eV) and CsPbBr_3_ (2.3 eV) with prominent phase stability were used in PSCs. Till now, CsPbIBr_2_‐based PSCs have yield a high PCE of 10.8% reported by our group.^[^ ^13^ ^]^ Intriguingly, PSCs constructed with CsPbBr_3_ have achieved a PCE over 10%.^[^ ^14^ ^]^ But its abjective wide bandgap hinders the further optimization owing to the Shockley–Queisser limit. Above all, I‐rich inorganic perovskites can deliver high PCE for PSCs with instability, whereas Br‐rich perovskite‐based devices with excellent stability usually exhibit a poor PCE. Up to now, extensive efforts have been exerted to promote performance of inorganic PSCs, including optimization of transport layers,^[^ ^15^, ^16^ ^]^ additive engineering,^[^ ^17^, ^18^ ^]^ interface modification,^[^ ^19^, ^20^, ^21^, ^22^ ^]^ and composition engineering.^[^ ^10^, ^23^, ^24^ ^]^ Beyond the aforementioned strategies, crystallization optimization is of excellent and universal availability for various categories of perovskites because outstanding crystallization is a perquisite to ensure the photovoltaic performance. Currently, some useful strategies have been implemented to regulate the crystallization of inorganic perovskites, including intermolecular exchange,^[1c]^ cation exchange,^[^ ^25^ ^]^ introducing steric hindrance,^[^ ^26^ ^]^ antisolvent engineering,^[^ ^27^ ^]^ warm spin coating,^[^ ^28^ ^]^ illumination‐promoted crystallization,^[^ ^29^ ^]^ and twice‐coating deposition.^[^ ^30^ ^]^ However, these strategies can complicate the fabrication procedures and be arduously extended to different inorganic perovskites due to the limitation deriving from the modification mechanism.

Inducing crystallization in nanoconfined space is a novel universal approach to control crystallization behavior, which can modulate crystallinity, phase, orientation, and grain size.^[^ ^31^, ^32^ ^]^ To date, anodized aluminum oxide (AAO) is the most widely used mesoporous scaffolds to form nanoconfined space. Kwon et al. demonstrated that 1D perovskite nanoarrays grown in AAO possess faster charge transport/extraction characteristics, lower defect density, and larger lattice strain than the bulk perovskite in planar structure.^[32a]^ Waleed et al. prepared 3D vertical CsPbI_3_ nanowires (NWs) inside AAO and discovered that the as‐grown NWs have stable cubic phase at room temperature owing to the effective encapsulation by AAO and large specific area of NWs, which display decent performance in photodetector applications.^[^ ^33^ ^]^ However, the deposition of AAO on substrates involves numerous complex processes, including deposition, electrochemical etching, and oxidizing annealing, which hinder the application in further developments.

To our knowledge, the easily obtainable mesosilica family covering SBA‐15, MCM‐41, KIT‐6, MSU‐H, and MCM‐48 has been incorporated in perovskite nanocrystals to benefit photoluminescence (PL) performance due to the nanoconfined crystallization effect.^[^ ^34^, ^35^, ^36^ ^]^ However, this appealing strategy has not been applied in the photovoltaic conversion field because the size of the aforementioned mesosilica is considerably large for the thin perovskite film, and the nanofluidic effect will impede the insertion of perovskite precursors.^[^ ^37^ ^]^ Therefore, we synthesized the Zr‐doped SBA‐15 (named as ZrSBA‐15) nanoplatelets and applied it in inorganic PSCs because it possesses similar pore structure but suitable size compared with the other mesoporous silica materials. Different amounts of ZrSBA‐15 were added in the perovskite precursors to prepare nanoconfined inorganic perovskite films where both CsPbIBr_2_ and CsPbI_3_ perovskites were utilized. Transmission electron microscopy (TEM) confirmed the nanoconfined crystallization of perovskites in ZrSBA‐15. Meanwhile, X‐ray diffraction (XRD) analysis demonstrated the lattice expansion and enlarged lattice strain. As such, the semiconductor properties of inorganic perovskite layers were improved, including increased charge transportation and suppressed nonradiative recombination. As expected, PSCs based on the modified inorganic perovskite layer exhibited superior PCE and stability to the pristine sample. Thus, this study proposed a facile strategy of introducing nanoconfined crystallization process to enhance the photovoltaic performance of inorganic PSCs.

## 2 Results and Discussion

**Figure 1:**
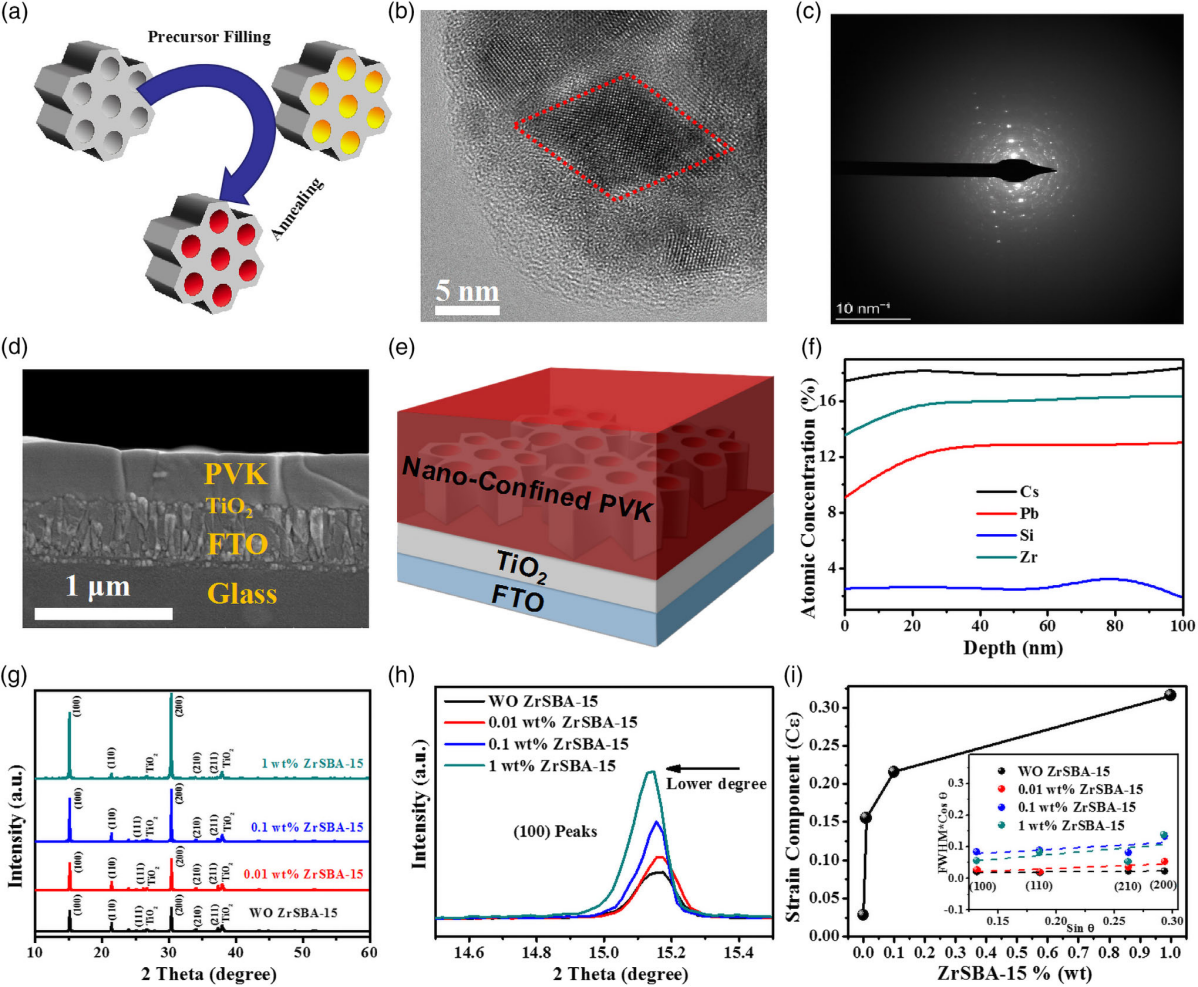
a) Schematic diagram of the nanoconfined crystallization of CsPbIBr_2_ in ZrSBA‐15; b) high‐resolution TEM image of CsPbIBr_2_ film with 0.1 wt% ZrSBA‐15; c) SAED of the nanoconfined crystals; d) cross‐sectional SEM of CsPbIBr_2_ film; e) schematic diagram of CsPbIBr_2_ film with ZrSBA‐15; f) atomic concentration versus film thickness extracted from XPS; g) XRD patterns of various CsPbIBr_2_ films with different amount of ZrSBA‐15; h) zoomed‐in (100) peaks; and i) strain component calculated from the slope of internal Williamson–Hall plots.

**Figure 2:**
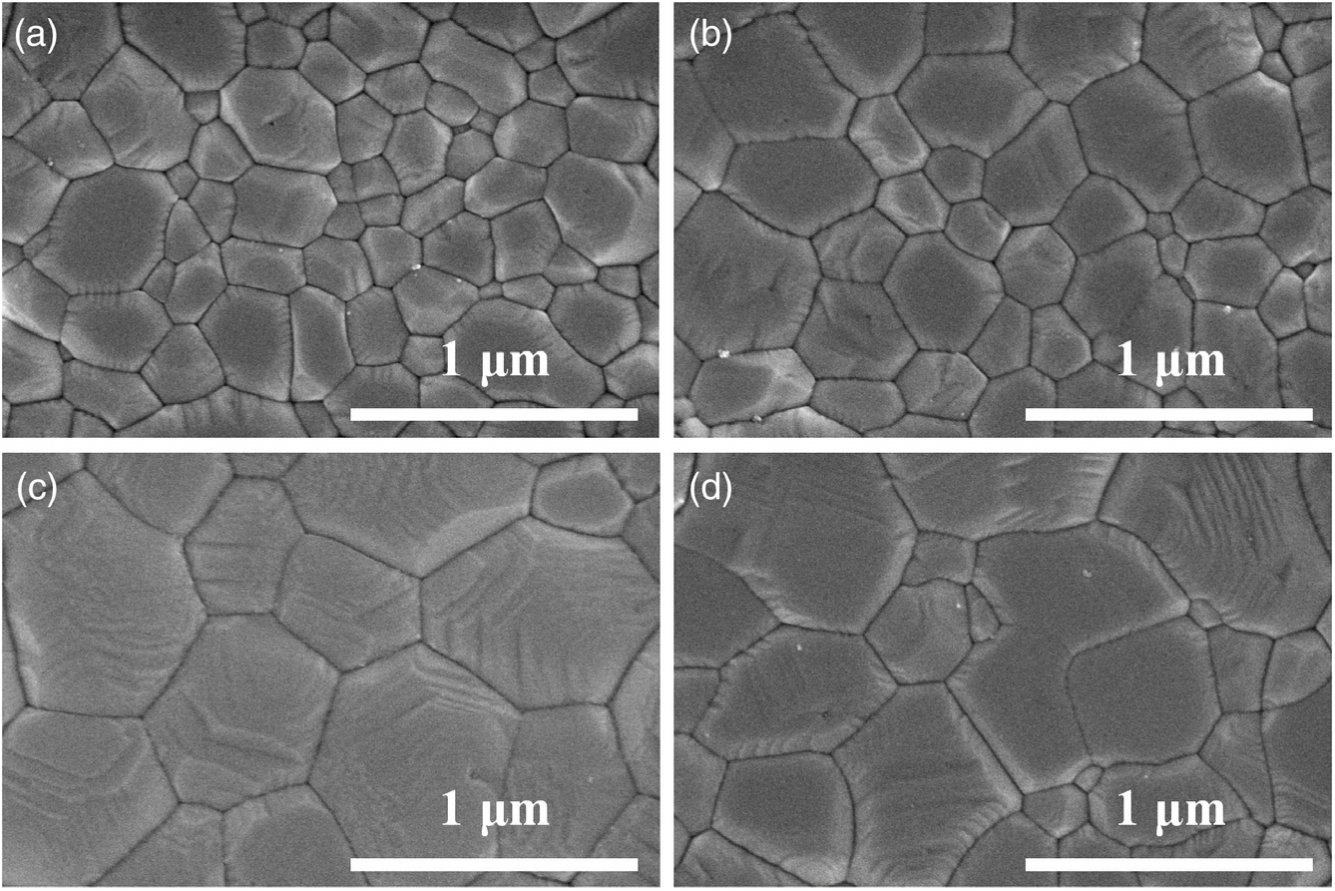
Top‐view SEM images of various CsPbIBr_2_ perovskite films: a) without ZrSBA‐15; b) with 0.01 wt% ZrSBA‐15; c) with 0.1 wt% ZrSBA‐15; and d) with 1 wt% ZrSBA‐15.

ZrSBA‐15 was synthesized by using the hydrothermal method and examined via a series of analyses. Figure S1a–c, Supporting Information, shows that ZrSBA‐15 is composed of hexagonal nanoplatelets with the thickness of 200 nm and the radius exceeding 1 μm, which also possesses 1D ordered mesopores. From the Brunauer–Emmett–Teller (BET) analyses in Figure S1d,e, Supporting Information, an average pore size of 6.92 nm and high specific surface area of 732.50 m^2^ g^−1^ can be calculated by using the Barrett–Joyner–Halenda (BJH) model. XRD result in Figure S1f, Supporting Information, indicates that ZrSBA‐15 is amorphous. In this work, trace amount of ZrSBA‐15 powder was incorporated into inorganic perovskite precursors to form a dispersion. The microfluidics state of precursor inserted in ZrSBA‐15 mesopores was simulated by injecting precursor in a quartz tubule. As per Figure S2a, Supporting Information, the liquid level in quartz tubule is identified as a concave level. The successfully filling of precursor in ZrSBA‐15 pores can be confirmed by Young–Laplace Equation (1)

(1) $$P_{S}=\gamma (\frac{1}{R_{1}}+\frac{1}{R_{2}})$$

where *P*_S_ is the received resultant force of liquid level, *γ* represents the interface tension, and *R*_1_ and *R*_2_ refer to the radius of curvature, respectively. Figure S2b, Supporting Information, depicts the curvature radius of discretionary liquid level. Due to the concave level, *R*_1_ equals to *R*_2_ and both are negative value. As a result, the direction of *P*_S_ always points to the side without liquid based on Young–Laplace theory; in other words, the precursor can effectively fill in the pores. Similarly, to reveal the contact status of perovskite precursor on ZrSBA‐15, a quartz plate was utilized as substrate to observe the contact angle, as per Figure S2c, Supporting Information, which is less than 90^o^. The spontaneous filling of precursor in ZrSBA‐15 pores can also be determined according to Equation (2) which is the derivation of Laplace equation based on the liquid level in tubule

(2) $$h=\frac{2\gamma \cos\theta }{R\rho g}$$

where *h* is the height of liquid level, *θ* is the contact angle, *R* is the radius of curvature, *ρ* is the precursor density, and *g* represents the gravitational acceleration. Due to the *θ* less than 90^o^, the value of *h* will always keep positive, thus demonstrating that the perovskite precursor can spontaneously fill in the pores. Therefore, we can rationally infer that perovskite can crystallize inside the pores of ZrSBA‐15 after annealing, as shown in **Figure** **1**a. To testify this conjecture, TEM measurement was performed on 0.1 wt% ZrSBA‐15 involved CsPbIBr_2_ perovskite, as shown in Figure S3a, Supporting Information, where the numerous spots represent the perovskite insertion in ZrSBA‐15. Correspondingly, the high‐resolution image in Figure 1b also exhibits the perovskite nanocrystal of around 7 nm, in line with the pore size of ZrSBA‐15. In addition, selected area electron diffraction (SAED) in Figure 1c demonstrates that this perovskite nanocrystal exists as quasi‐single crystals and the corresponding selected area can be found in Figure S3c, Supporting Information. On the contrary, the SAED of whole crystal displays a circle diffraction of ordinary polycrystalline features as per Figure S3d, Supporting Information, and the corresponding selected area can be found in Figure S3b, Supporting Information. As such, we can conclude that the 1D mesopores of ZrSBA‐15 induced the nanoconfined crystallization of perovskite and continuously fostered the formation of 1D perovskite monocrystal. In addition, the cross‐sectional scanning electron microscopy (SEM) image of CsPbIBr_2_ perovskite film in Figure 1d demonstrates the film thickness is around 250 nm. By converging the smooth top‐view SEM images in **Figure** **2** and the morphology of ZrSBA‐15 nanoplatelets, we deduced ZrSBA‐15 must be laid flatly on the substrates as shown in Figure 1e and then 1D perovskite monocrystal vertically exists in the thin film. Apart from the nanoconfined crystallization effect, X‐ray photoelectron spectroscopy (XPS) was utilized to investigate the chemical interplay between ZrSBA‐15 and CsPbIBr_2_ perovskite crystals. As shown in Figure S4, Supporting Information, all the elements display negligible peak variation, indicating that there exists no chemical interplay between perovskite and ZrSBA‐15. In addition, all the elements homogeneously distribute in the ZrSBA‐15‐incorporated CsPbIBr_2_ perovskite film according to XPS depth profiles in Figure 1f.

To further investigate the effect of nanoconfined crystallization, XRD analysis was performed on CsPbIBr_2_ perovskite films with different amount of ZrSBA‐15. In Figure 1g, four diffraction peaks appear at 15.05°, 21.34°, 30.16°, and 33.91° in all samples, which can be assigned to the (100), (110), (200), and (210) planes of CsPbIBr_2_; in addition, other peaks of CsPbIBr_2_ and substrate are directly labeled in Figure 1g. Upon increasing the amount of ZrSBA‐15, the peak intensities of (100) and (200) planes increased substantially, indicating the enhanced crystallinity. Meanwhile, the intensity of other peaks fades out along with the introduction of ZrSBA‐15, which attests a considerably powerful preferred orientation of <100>.

Zoomed‐in diffraction peaks of (100) plane in Figure 1h show the shifts toward a lower angle, suggesting the lattice expansion after introducing ZrSBA‐15. Naturally, the lattice deformation ought to influence the lattice strain, which was further analyzed qualitatively by using strain component variation in Figure 1i, according to the slope of Williamson–Hall plot (inset of Figure 1i). Evidently, the lattice strain in CsPbIBr_2_ increases substantially with the addition of ZrSBA‐15. It is worth noting that such mechanism of strain variation is individual in comparison with the case of additive engineering. The latter is associated with the insertion or substitution of extrinsic ions, whereas the former, in the current study, is attributed to the tensile stress between the inner walls of ZrSBA‐15 and perovskite crystals while constraining the shrinkage during crystallization.[^31^, ^38^]

Figure 2 and S6, Supporting Information, are the top‐view SEM images of CsPbIBr_2_ perovskites with various amount of ZrSBA‐15. Compared with the pristine one, no evident variation in surface morphology in addition to the enlarged grains was observed for the films with 0.01 and 0.1 wt% ZrSBA‐15 incorporation. All of them possess dense and compact morphologies with a high coverage (Figure S6a–c, Supporting Information). By contrast, the sample with 1 wt% ZrSBA‐15 deviated significantly from the others as evidenced in Figure S6d, Supporting Information, where the numerous large pinholes indicate that the excessive ZrSBA‐15 may disrupt the homogeneous formation of high‐quality thin films. Figure S5, Supporting Information, depicts the grain size distributions, indicating that perovskite film with 0.1 wt% ZrSBA‐15 possesses the largest grain size with least grain boundaries. Meanwhile, from the atomic force microscopy (AFM) in Figure S7, Supporting Information, root‐mean‐square is reduced from 47.3 to 32.1 nm with the assist of ZrSBA‐15, thereby leading to excellent stability.

As mentioned, unlike the other additives, ZrSBA‐15 only induces nanoconfined crystallization and has no interplay with CsPbIBr_2_ perovskite. The mechanism of enlarging grain size by introducing ZrSBA‐15 is discussed as followed. On one hand, the specific surface area (*A*) of precursor is largely increased due to the high specific surface area of ZrSBA‐15, and then the entropy of crystallization (*S*) is enlarged according to Maxwell relationship between interfacial tension and temperature (3)

(3) $$\frac{\partial S}{\partial A}=-\frac{\partial \gamma }{\partial T}$$

(4) dG=dH−TdS

where *T* is the reaction temperature. Combining with Equation (4), where *G* is the Gibbs free energy and *H* is the enthalpy, as a result, it is deduced that the increased *A* leads to a reduced Gibbs free energy of crystallization, thereby assisting the nucleation process. On the other hand, according to the contact angle measurement in Figure S2, Supporting Information, it is inferred that the wettability of precursor on ZrSBA‐15 is inferior to that of TiO_2_. Previous result has demonstrated that a nonwetting surface suppresses heterogeneous nucleation and yields a higher grain boundary mobility, thus resulting in less dense nuclei and larger grain size.^[^ ^39^ ^]^ In addition, according to Kelvin Equation (5)

(5) $$RT\ln(p_{2}/p_{1})=\frac{2\gamma M}{\rho }(\frac{1}{r_{2}}-\frac{1}{r_{1}})$$

where *R* is the molar gas constant, *M* is the molar mass of precursor, *p*_1_ and *p*_2_ are saturated vapor pressure of precursor on TiO_2_ and quartz plate, and *r*_1_ and *r*_2_ denote the curvature radius of perovskite precursor on TiO_2_ and quartz plate, respectively. A smaller curvature radius reflected by the larger contact angle results in an enlarged saturated vapor pressure, which eventually facilitates the volatilization of solvent and thus promotes the crystallization.

**Figure 3:**
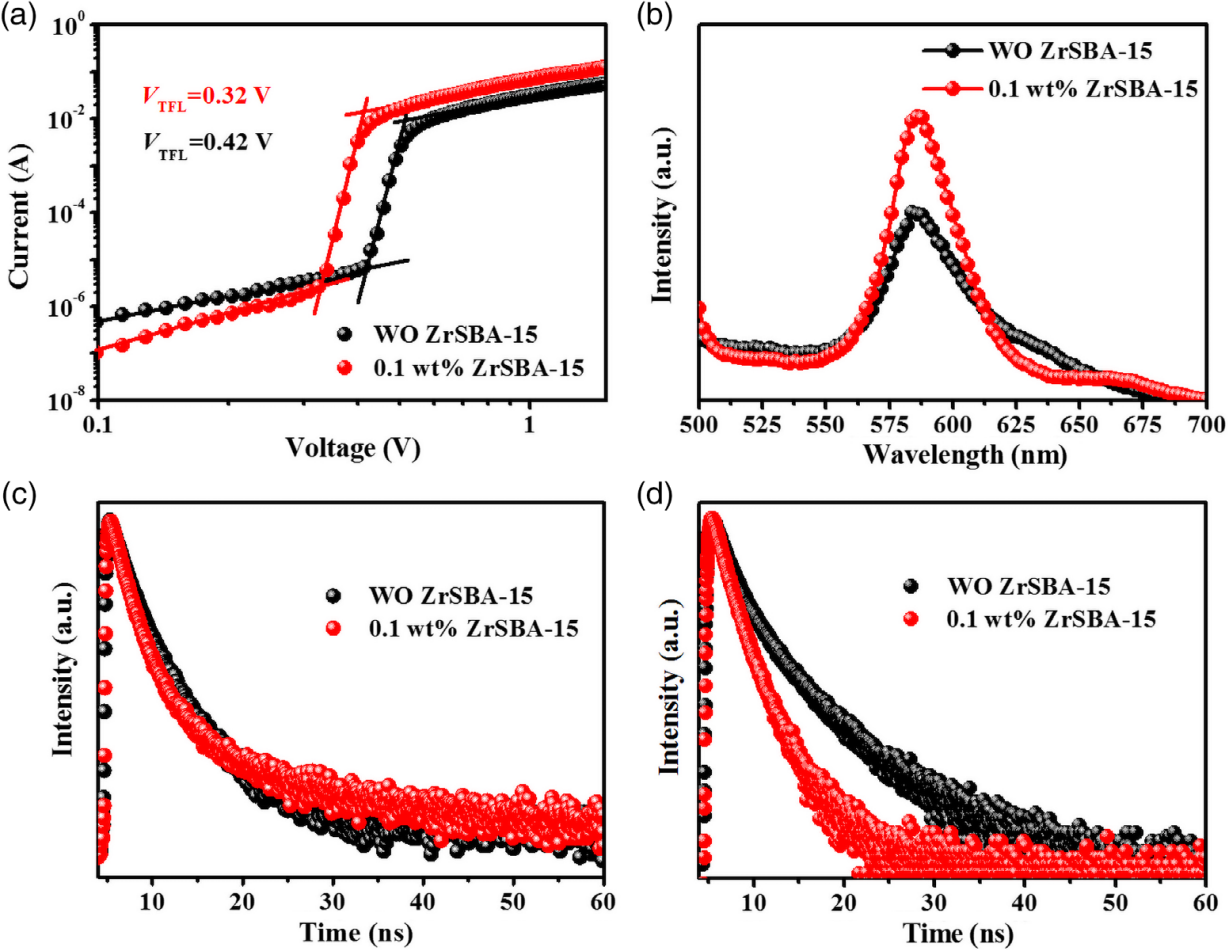
a) Dark current–voltage curves of the electron‐only devices with the structure of FTO/TiO_2_/CsPbIBr_2_/PCBM/Ag; b) steady‐state PL spectra motivated from perovskite side; c) time‐resolved PL spectra motivated from perovskite side; and d) time‐resolved PL spectra motivated from FTO/TiO_2_ side.

The variations of crystallinity, lattice strain, and morphology will influence the trap‐state density. **Figure** **3**a shows the dark current–voltage curves of electron‐only CsPbIBr_2_ devices with a configuration of FTO/TiO_2_/perovskite film/phenyl‐C_61_‐butyric acid methyl ester (PCBM)/Ag.^[^ ^40^ ^]^ The low potential region is identified as the Ohmic response due to the linear current–voltage relationship. With continuously increasing bias voltage, the pristine linear relationship is considerably turned into nonliner response, and the kink points are regarded to be the trap‐filled limited voltage (*V*_TFL_). The relationship between the trap‐state density (*n*_t_) and *V*_TFL_ is depicted in Equation (6)

(6) $$n_{t}=\frac{2V_{\text{TFL}}\varepsilon \varepsilon_{0}}{eL^{2}}$$

where *e* represents the charge of an electron, *L* is the perovskite film thickness (250 nm), *ε* refers to the dielectric constant of CsPbIBr_2_, and *ε*_0_ is the vacuum permittivity. The calculated *n*_t_ of the 0.1 wt% ZrSBA‐15‐incorporated CsPbIBr_2_ perovskite is 4.53 × 10^15 ^cm^−3^, which is evidently decreased compared with that (i.e., 6.10 × 10^15^ cm^−3^) of the control sample. In theory, the trap‐state density should be increased because the nanoconfined crystallization brings in more grain boundaries accompanied with surface defects at the ZrSBA‐15/perovskite interface, but fortunately, it was compensated by the enlarged crystal grain and the trap‐state density is reduced eventually. In addition, electron mobility (*μ*) can be extracted from the trap‐filled region according to Equation (7)

(7) $$J_{D}=\frac{9\varepsilon \varepsilon_{0}\mu V_{b}^{2}}{8L^{3}}$$

where *J*_D_ is the dark current and *V*_b_ is the applied voltage. The calculated carrier mobility of the 0.1 wt% ZrSBA‐15‐incorporated CsPbIBr_2_ perovskite is 9.51 × 10^−3^ cm^2^ (V s)^−1^, which is superior to that (i.e., 2.69 × 10^−3^ cm^2^ (V s)^−1^) of the pristine one. The increased electron mobility must be in line with the nanoconfined 1D perovskite single crystal structure and the enhanced grain size upon incorporating ZrSBA‐15.

Furthermore, PL spectra of CsPbIBr_2_ films without and with 0.1 wt% ZrSBA‐15 were utilized to study the charge recombination at the interfaces and in the bulk perovskite. The device structure of glass/TiO_2_/CsPbIBr_2_ was adopted because the crystallization of CsPbIBr_2_ is negatively influenced by the bare glass surface. Given that the absorption depth in perovskite is estimated to be shorter than the film thickness, we considered that the PL result can depict the charge dynamic process in perovskite layers and disregard TiO_2_ when the sample is irradiated from the perovskite side. Figure 3b shows the steady‐state PL spectra motivated from the perovskite side. In addition to the similar emission peak position, the peak intensity of the ZrSBA‐15‐incorporated perovskite is considerably higher than that of the control sample. As shown in Figure 3c, the corresponding time‐resolved PL spectrum of CsPbIBr_2_ film with 0.1 wt% ZrSBA‐15 exerts a slightly prolonged average life time of 3.09 ns compared with the 2.66 ns of the control film. This result attests that CsPbIBr_2_ perovskite with 0.1 wt% ZrSBA‐15 enjoys fewer defects and effectively suppressed nonradiative recombination in comparison with the pristine sample. Time‐resolved PL spectra measured from the glass/TiO_2_ side were utilized to inspect the charge extraction dynamics. As per Figure 3d, the carrier life time is reduced from 3.30 to 1.74 ns upon incorporating 0.1 wt% ZrSBA‐15. We deduced that the significantly accelerated charge extraction process is benefited from the 1D perovskite single crystals in the nanoconfined pores, which acts as highways for electron transportation.

**Figure 4:**
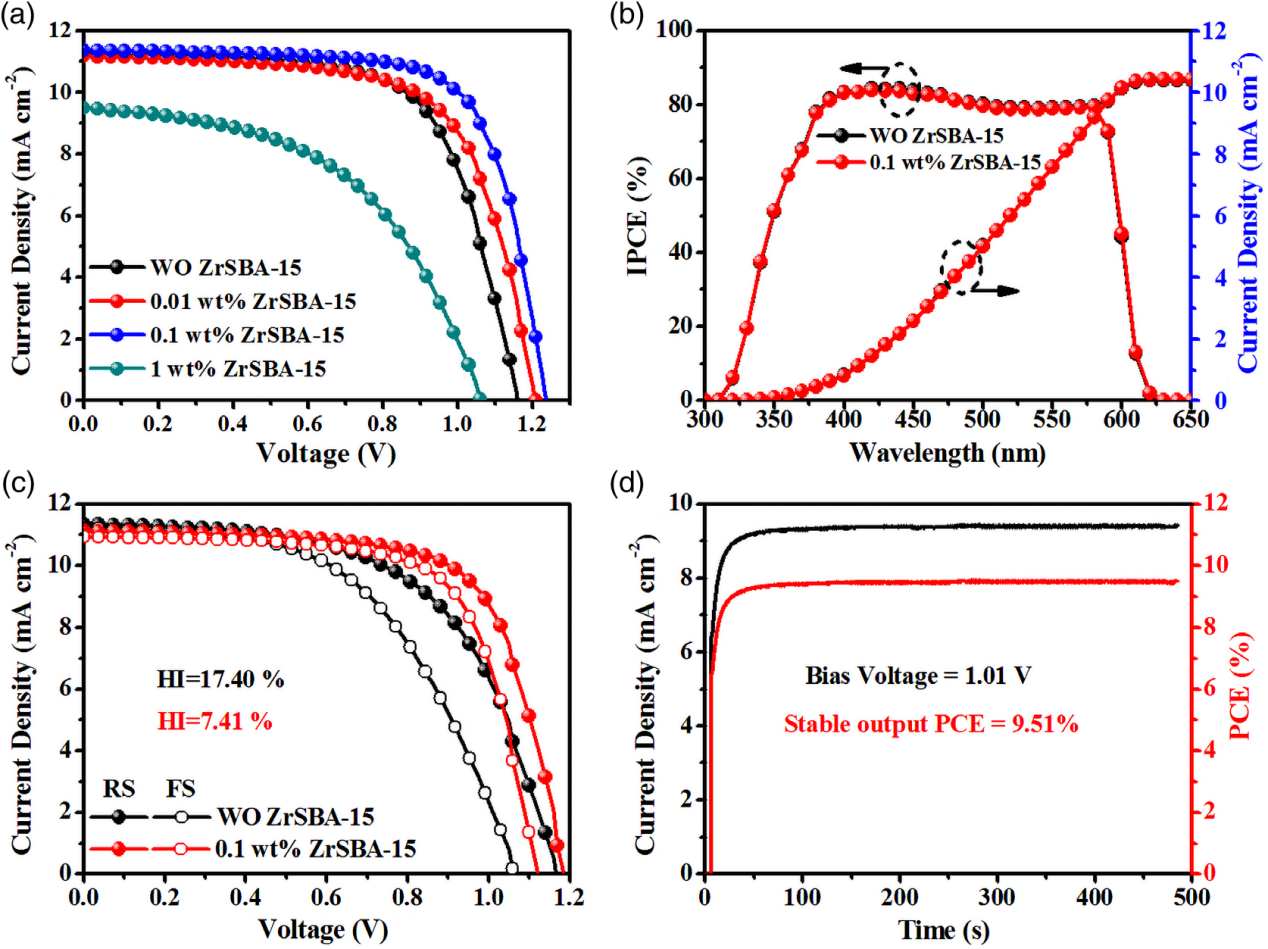
a) *J*–*V* curves of champion CsPbIBr_2_ PSCs with various amount of ZrSBA‐15; b) IPCE plots of CsPbIBr_2_ PSCs without and with 0.1 wt% ZrSBA‐15; c) reverse and forward scanning *J*–*V* curves of CsPbIBr_2_ PSCs without and with 0.1 wt% ZrSBA‐15; and d) stabilized power output of CsPbIBr_2_ PSCs with 0.1 wt% ZrSBA‐15.

To study the dependence of device performance on nanoconfined crystallization, PSCs with a planar structure of FTO/TiO_2_/CsPbIBr_2_/Spiro‐OMeTAD/Au were fabricated and examined under AM1.5G solar illumination at 100 mW cm^−2^. Device performance distribution with 60 counts is shown in Figure S8a and Table S1, Supporting Information. **Figure** **4**a shows the champion *J*–*V* curves of PSCs with different amounts of ZrSBA‐15. By increasing the amount of ZrSBA‐15 from 0 to 0.1 wt%, the open‐circuit voltage (*V*_OC_) and fill factor (FF) of devices are increased notably, which can be ascribed to the minimal trap‐state density and enhanced charge transportation. By contrast, the excess ZrSBA‐15 of 1 wt% can extremely degrade the device performance due to the unfavorable morphology as reflected by the poor absorption capability (Figure S9a, Supporting Information). Eventually, the optimal CsPbIBr_2_ PSCs with 0.1 wt% ZrSBA‐15 yield a champion PCE of 10.04% along with a *V*_OC_ of 1.23 V. Comparing with the pristine, the short‐circuit current density (*J*_SC_) is constant, regardless of the introduction of ZrSBA‐15, which is further confirmed by the incident photon‐to‐current conversion efficiency (IPCE) in Figure 4b and bandgap in Figure S9b, Supporting Information. An impressive external quantum efficiency of 80% in the wavelength region from 300 to 600 nm is attained. The integrated current density (10.41 mA cm^−2^) from IPCE is almost consistent with the *J*_SC_, while the small discrepancy is ascribed to slow charge dynamics and spectral mismatch between the IPCE source and the solar simulator.

Hysteresis, as a notorious phenomenon in PSC current–voltage performance, is triggered by ion migration, carrier dynamics at interface, and deeper trap effects in perovskite films.^[^ ^41^, ^42^, ^43^ ^]^ To our knowledge, mixed halide perovskite suffers from severe phase segregation induced by ion migration under illumination, which will, in turn, aggravate hysteresis in PSCs.^[^ ^44^, ^45^, ^46^, ^47^, ^48^, ^49^ ^]^ Therefore, mitigating hysteresis in PSCs constructed with inorganic Br‐rich perovskite is of paramount importance. In this work, hysteresis index (HI) was used to judge hysteresis quantitatively, which is defined according to Equation (8)

(8) $$\text{HI}=\frac{\eta_{\text{RS}}-\eta_{\text{FS}}}{\eta_{\text{RS}}}$$

where *η*_RS_ and *η*_FS_ represent PCEs obtained from the reverse and forward scans, respectively. As shown in Figure 4c, the HI of CsPbIBr_2_ PSCs is decreased from 17.4% to 7.4% after the introduction of 0.1 wt% ZrSBA‐15, which can be ascribed to the defect passivation and promoted charge transportation caused by 1D perovskite monocrystal. However, the HI of optimized PSCs still remains 7.4%, which can be attributed to the migration ions piled up at the interface between perovskite and TiO_2_, producing larger injection barriers, hampering electron extraction, and leading to strong current density–voltage hysteresis in the mixed halide inorganic perovskite solar cells.^[^ ^44^ ^]^ Therefore, CsPbIBr_2_ PSCs still suffer from a 7.4% HI although after optimization. Despite reduced HI, the nonignorable hysteresis behavior can lead to an overestimate of PCE and elicit concerns about the actual power output. Therefore, stabilized power output (SPO) was measured on the 0.1 wt% ZrSBA‐15‐based CsPbIBr_2_ PSCs. In Figure 4d, a steady PCE of 9.51% can be maintained for exceeding 450 s with a steady current density of 9.42 mA cm^−2^ under ambient atmosphere. Stability test was conducted in an ambient environment with a relative humidity of 35%. CsPbIBr_2_ PSCs with 0.1 wt% ZrSBA‐15 exhibit a lower degradation rate than the control sample. The former can retain 91% of its initial PCE after aging for 140 h (see Figure S10a–f, Supporting Information). This improved stability may be related with the large grain size and enhanced crystallinity of perovskites.

We considered that such a nanoconfined crystallization strategy is universal to various perovskites. Therefore, ZrSBA‐15 was also successfully applied in the CsPbI_3_ PSCs. Figure S8b, Supporting Information, shows the *J*–*V* curves of CsPbI_3_ PSCs without and with 0.1 wt% ZrSBA‐15, which confirms an enhanced PCE from 14.10% to 14.69% accompanied by an increased *V*_OC_ from 0.95 to 1.03 V after incorporating ZrSBA‐15. This similar improving tendency further demonstrates that the nanoconfined crystallization effect of ZrSBA‐15 can universally tailor the property of inorganic perovskite and continuously lead to enhanced photovoltaic performance.

**Figure 5:**
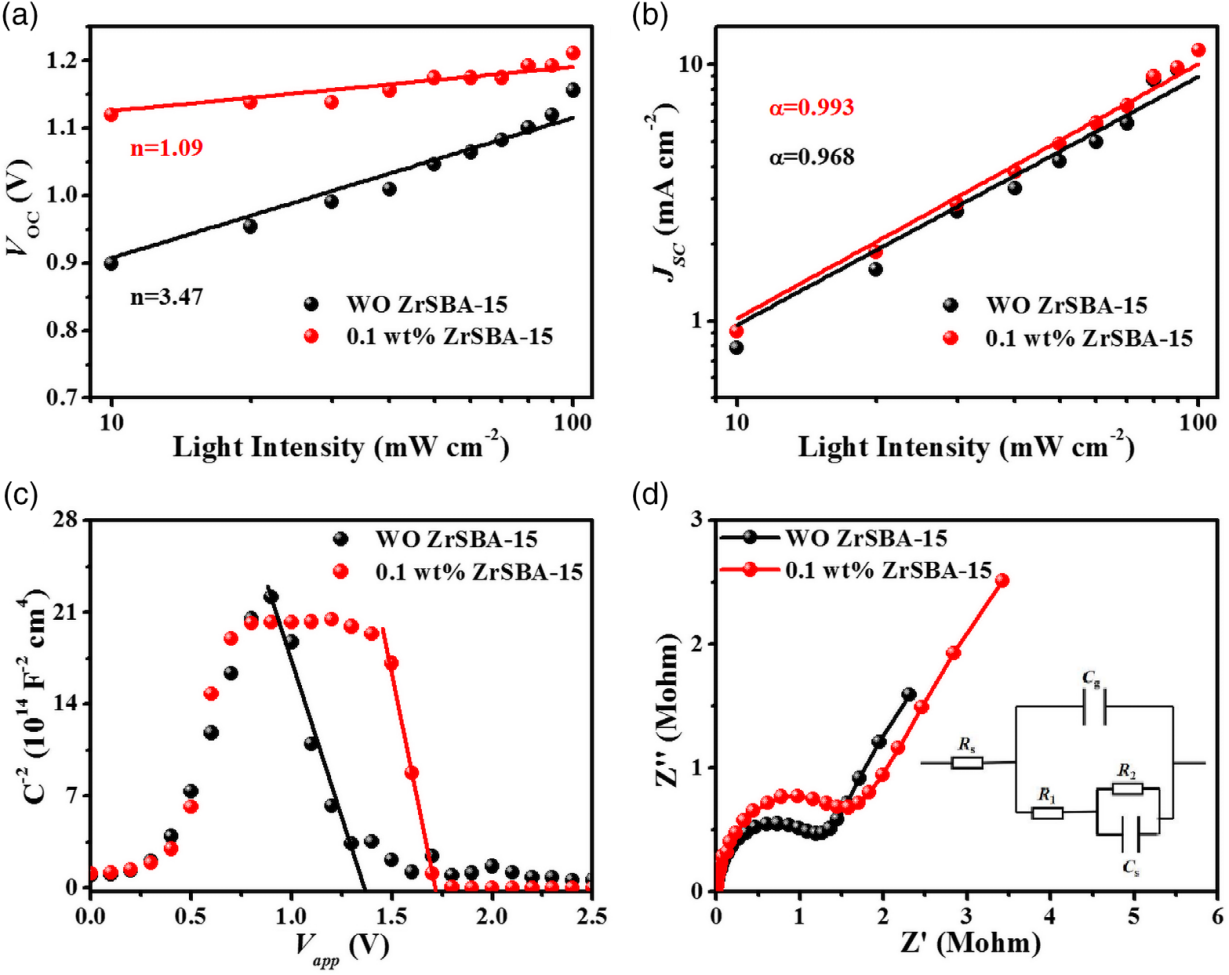
a) Light intensity dependent *V*_OC_ of CsPbIBr_2_ PSCs without and with 0.1 wt% ZrSBA‐15; b) Light intensity dependent *J*_SC_ of CsPbIBr_2_ PSCs without and with 0.1 wt% ZrSBA‐15; c) Mott–Schottky curves of CsPbIBr_2_ PSCs without and with 0.1 wt% ZrSBA‐15; and d) Nyquist plots of CsPbIBr_2_ PSCs without and with 0.1 wt% ZrSBA‐15.

To completely inspect the effect of ZrSBA‐15 on the CsPbIBr_2_ devices, the dependence of *V*_OC_ and *J*_SC_ on light intensity was tested to investigate the charge recombination in PSCs. The relationship between light intensities (*I*) and the corresponding *V*_OC_ can be described through Equation (9)

(9) $$\frac{dV_{\text{OC}}}{d\mathrm{lg}I}=2.303\frac{nk_{B}T}{e}$$

where *n*, *k*_B_, and *T* represent the ideal factor, Boltzmann constant, and thermodynamic temperature, respectively. The ideal factor equaling to 1 is regarded as a low‐level Shockley–Read–Hall (SRH) recombination in parallel with minimal trap‐state density. In **Figure** **5**a, the calculated *n* of pristine and ZrSBA‐15‐based CsPbIBr_2_ PSCs is 3.47 and 1.09, respectively. Therefore, the trap‐assisted SRH recombination was relatively suppressed upon the incorporation of ZrSBA‐15.^[^ ^50^ ^]^ Figure 5b shows the light intensity versus *J*_SC_ based on power law *J*_SC_ ∝ *I* ^*α*^, indicating that the ZrSBA‐15‐based CsPbIBr_2_ PSCs possess negligible bimolecular recombination and substantially charge extraction due to the value of *α* closer to 1 when comparing with the control PSCs.^[^ ^51^, ^52^ ^]^

Electrochemical characterizations were implemented to investigate the effects of ZrSBA‐15 on the charge dynamic processes. The Mott–Schottky curves are plotted in Figure 5c to estimate the built‐in potential and explore the origin of the difference in *V*_OC_. The built‐in potential can be obtained from the intercept at the horizontal axis, according to Equation (10)

(10) $$\frac{1}{C^{2}}=\frac{2}{e\varepsilon \varepsilon_{0}N_{D}}(V_{\text{bi}}-V-\frac{kT}{e})$$

where *C*, *V*_bi_, *V*, and *N*_D_ are the capacitance of the space charge area, built‐in potential, applied voltage, and donor density, respectively. The fitted built‐in potential of CsPbIBr_2_ PSCs with 0.1 wt% ZrSBA‐15 is 1.71 V, which is significantly superior to the 1.36 V of the pristine sample. The enhanced built‐in potential indicates an increased driving force for charge transport and broadened depletion region for recombination suppression, which are positive for leading to an enhanced *V*_OC_.^[^ ^53^ ^]^ Electrochemical impedance spectroscopy (EIS) was further performed to track the charge‐transfer process in PSCs. Figure 5d shows the Nyquist plots of CsPbIBr_2_ PSCs without and with 0.1 wt% ZrSBA‐15. The internal equivalent circuit was utilized for EIS fitting, where *R*_s_ represents the series resistance related to the wire and circuit contact, *C*_g_ signifies the geometric capacitance of the bulk perovskite, *C*_s_ is the charge accumulation capacitance with respect to the perovskite contact interface, and *R*_1_ and *R*_2_ are the recombination resistance (*R*_rec_ = *R*_1_ + *R*_2_) because the direction of bias voltage is opposite to that of the charge transportation under operating condition. The *R*_rec_ as a function of bias voltages from 0.1 to 0.4 V is plotted in Figure S11a, Supporting Information, where the increased *R*_rec_ attesting the charge recombination process is restrained after the introduction of ZrSBA‐15. Furthermore, the significantly decreased charge accumulation capacitance *C*_s_ shown in Figure S11b, Supporting Information, demonstrates the reduced surface charge accumulation, which is consistent with the mitigatory hysteresis in Figure 4c.

## 3 Conclusions

In summary, a novel and facile strategy was developed to achieve nanoconfined crystallization of inorganic perovskite, in which ZrSBA‐15 with 1D ordered mesopores was introduced into CsPbIBr_2_ perovskite. Nanoconfined crystallization leads to the formation of 1D perovskite monocrystal, facilitating the charge transport and extraction. Simultaneously, ZrSBA‐15 also benefits the crystallinity and morphology of perovskite, thereby further bringing down the defect density and enhancing the film stability. Consequently, PSCs based on ZrSBA‐15‐incorporated CsPbIBr_2_ exhibit a champion PCE of 10.04% compared with 8.67% of the pristine one. In addition, this nanoconfined crystallization engineering can be extended to CsPbI_3_ PSCs owing to the absence of chemical reactions, as reflected by the boosted PCE from 14.10% to 14.69% after using ZrSBA‐15. The initial introduction of mesosilica into PSCs in this work provides a new approach to tailor the crystallization process of perovskite and presents a universal method to improve the performance of inorganic PSCs.

## 4 Experimental Section

4.1

4.1.1

##### Chemicals

Cesium iodide (CsI), lead bromide (PbBr_2_), dimethylsulfoxide (DMSO), *N,N*‐dimethylformamide (DMF), acetonitrile, and chlorobenzene were purchased from Sigma‐Aldrich. HPbI_3_, Spiro‐OMeTAD, was purchased from Xi'an Polymer Light Technology Cory. Pluronic P123 was purchased from Adamas‐Beta. Titanium tetrachloride (TiCl_4_) was purchased from Aladdin. Zirconium oxychloride (ZrOCl_2_·8H_2_O) was purchased from Sinopharm Chemical Reagent. Ethyl alcohol, hydrochloric acid (HCl), and tetraethoxysilane (TEOS) were purchased from Tianjin Guangfu Fine Chemical Industry Research Institute.

##### Synthesis of ZrSBA‐15

First, 5 g of P123 (Pluronic) was dissolved in deionized water and 30 mL of 37% HCl was dropwise added under mild stirring for 30 min. Whereafter, 0.8 g of ZrOCl_2_·8H_2_O and 11 mL of TEOS were added in the mixed solution and stirred at 40 °C for 24 h. After fully reaction, the mixed solution was transferred into Teflon‐line autoclave and placed in an oven at 100 °C for 24 h. After crystallization, the filtered precipitate was washed with deionized water and ethanol for 3 times, respectively. The cleaned precipitate was dried at 80 °C and calcined at 550 °C for 5 h to remove the template.

##### Preparations of Precursors

PbBr_2_ (367 mg) and CsI (260 mg) were dissolved in 1 mL of DMSO to obtain the CsPbIBr_2_ perovskite precursor. HPbI_3_ (390 mg) and CsI (259 mg) were dissolved in 1 mL of DMF to obtain the CsPbI_3_ perovskite precursor. ZrSBA‐15 (2, 20, and 200 mg) was dispersed in 1 mL of DMSO to obtain the additive suspensions. The obtained suspensions were ultrasonically dispersed for 12 h before further utilization. Three additive suspensions (30 μL) were, respectively, added in 1 mL of perovskite precursors and final perovskite precursors were obtained with ZrSBA‐15 weight ratios of 0.01, 0.1, and 1 wt%.

##### Fabrication of Devices

FTO glass was ultrasonically washed with deionized water, ethanol, and isopropanol for 45 min, respectively. Compact TiO_2_ layer was chosen as electron transport layer and prepared as declared by a previous report.^[^ ^13^ ^]^ CsPbIBr_2_ perovskite precursors with 0, 0.01, 0.1, and 1 wt% ZrSBA‐15 were spin‐coated on TiO_2_ layer at 1500 rpm for 150 s, followed by annealing at 225 °C for 10 min. CsPbI_3_ perovskite precursors with 0 and 0.1 wt% ZrSBA‐15 were spin‐coated on TiO_2_ layer at 3500 rpm for 30 s, followed by annealing at 150 °C for 120 min. Spiro‐OMeTAD solution which was prepared according to a previous work was spin‐coated on perovskite layer as hole transport layer.^[^ ^17^ ^]^ All the spin‐coating operations were conducted in a glove box. Finally, 120 nm gold with active area of 0.09 cm^2^ was deposited on top of the as‐prepared devices by vacuum evaporation.

##### Characterizations

XRD patterns of various films were performed by Bruker D2 PHASER with Cu Kα irradiation (*λ* = 1.5406 Å). UV–vis adsorption spectra were recorded by U‐4150 Hitachi UV–vis spectrophotometer. SEM was operated on field‐emission SEM (Hitachi SU8020). BET analysis was described by JW‐BK222 analyzer. TEM characterizations were operated on field‐emission TEM (FEI Tecnai G2 F20). XPS spectra were tested by X‐ray photoelectron spectrometer Thermo Scientifc ESCALAB 250Xi. AFM images were characterized by Bruker Dimension Icon instrument. IPCE was measured on QE‐R Enlitech (R3018). The fluorescence spectra were measured on PicoQuant FT‐300 and FT‐100 with pulsed diode laser at 406.8 nm. The photocurrent density–voltage curves of PSCs were tested under AM1.5 G at 100 mW cm^−2^ (Newport Thermal Oriel 91 192). Various electrochemical characterizations were measured on Zahner, IM6e (Germany).

## Conflict of Interest

The authors declare no conflict of interest.

## Supporting information

Supplementary Material
