# Supplementary material for: Nanoconfined Crystallization for High‐Efficiency Inorganic Perovskite Solar Cells
Source: Small Sci. 2021 Jan 15;1(2):2000054. doi: 10.1002/smsc.202000054 (PMC11935812; doi:10.1002/smsc.202000054)
Supplement: Supplementary file 1 — Supplementary Material [file SMSC-1-2000054-s001.docx]

**Nano-Confined Crystallization for High Efficiency Inorganic Perovskite Solar Cells**

Xiao Jiang^a^, Kai Wang^a,*^, Hui Wang^a^, Lianjie Duan^a^, Minyong Du^a^, Likun Wang^a^, Yuexian Cao^a^, Lu Liu^a^, Shuping Pang^c^, Shengzhong (Frank) Liu^a,b,*^

^a^ *Dalian National Laboratory for Clean Energy; iChEM, Dalian Institute of Chemical Physics, Chinese Academy of Sciences, Dalian 116023, Liaoning, China; University of the Chinese Academy of Sciences, Beijing 100039, China*

^b^ *Key Laboratory of Applied Surface and Colloid Chemistry, Ministry of Education, Shaanxi Key Laboratory for Advanced Energy Devices, Shaanxi Engineering Lab for Advanced Energy Technology, Institute for Advanced Energy Materials, School of Materials Science and Engineering, Shaanxi Normal University Xi’an 710119, China*

^c^ *Qingdao Institute of Bioenergy and Bioprocess Technology, Chinese Academy of Sciences, Qingdao 266101, China*

^*^Corresponding authors.

1. mail: [szliu@dicp.ac.cn](mailto:szliu@dicp.ac.cn;), wangkai@dicp.ac.cn.


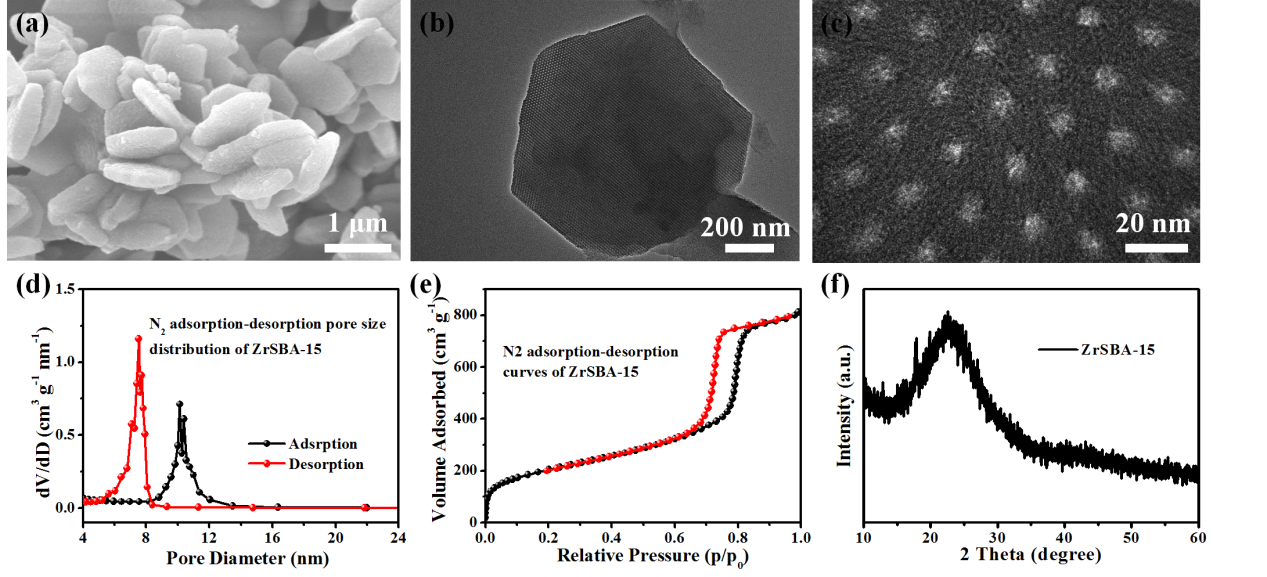


Figure S1. (a) SEM image of ZrSBA-15; (b), (c) TEM images of ZrSBA-15; (d) Pore diameter distributions of ZrSBA-15; (e) N_2_ adsorption-desorption curves of ZrSBA-15; (f) XRD pattern of ZrSBA-15.


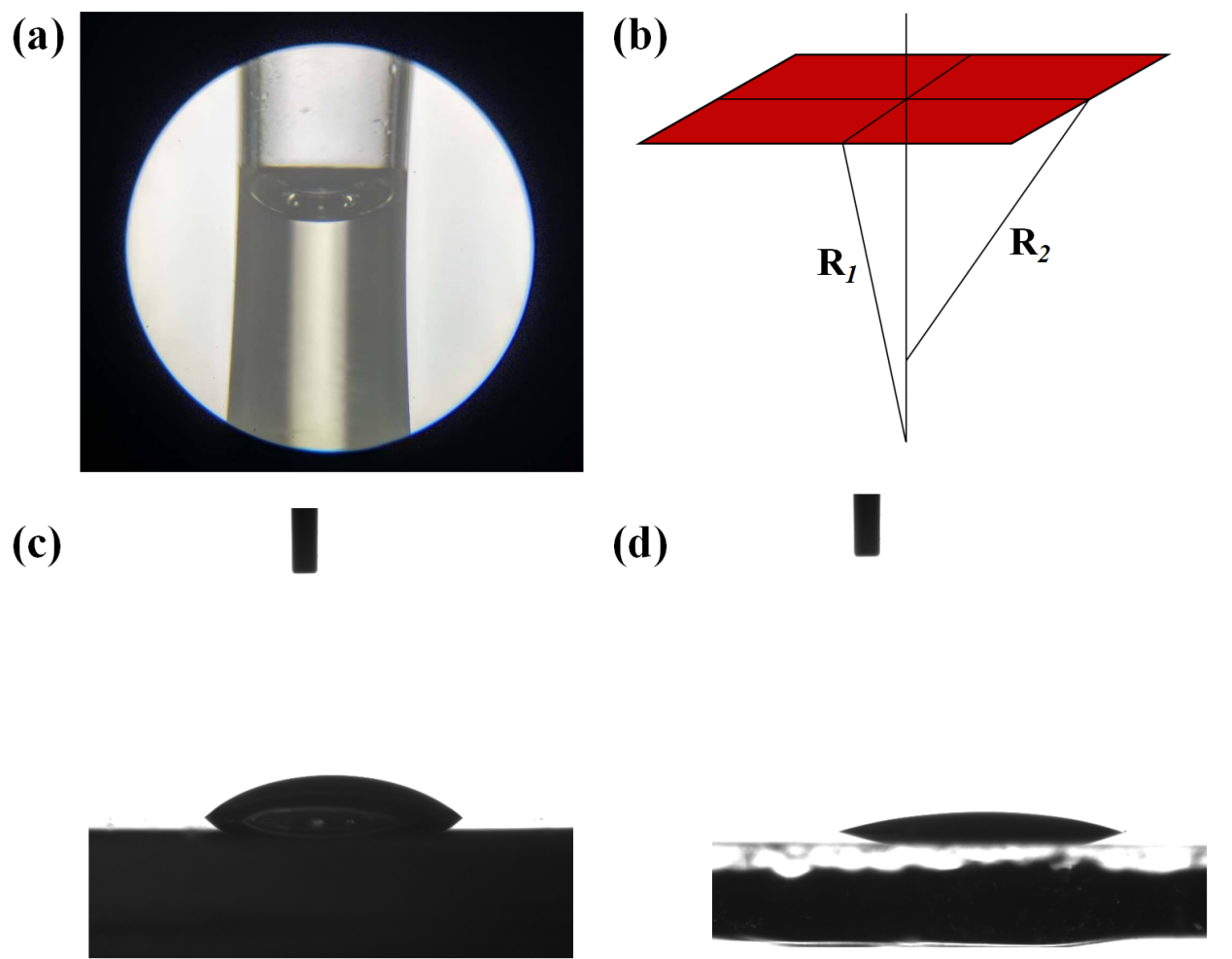


Figure S2. (a) Liquid level of perovskite precursor in quartz tubule; (b) Schematic diagram of discretionary liquid level in Young-Laplace equation; (c) Contact angle of perovskite precursor on quartz plate; (d) Contact angle of perovskite precursor on TiO_2_.


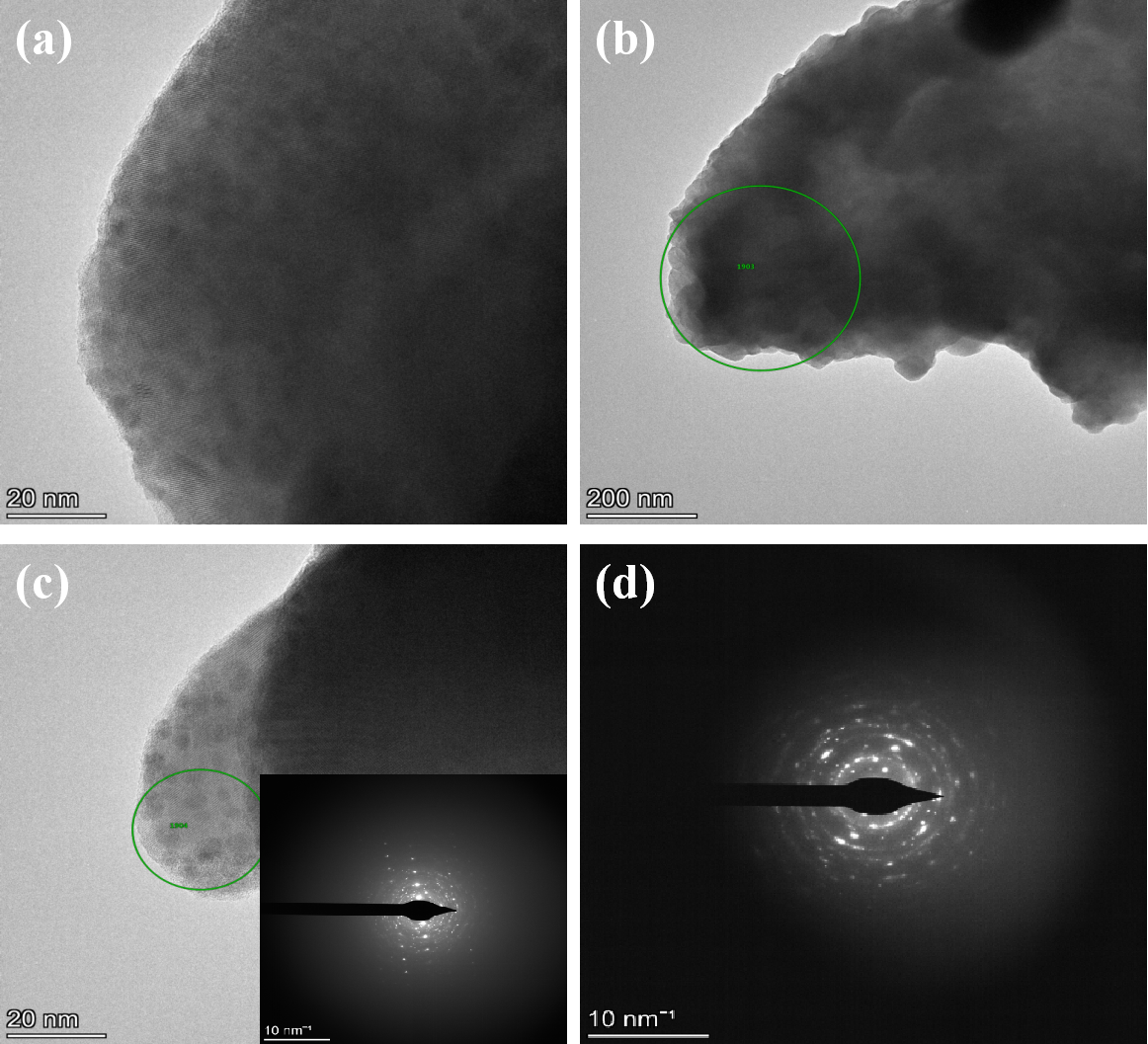


Figure S3. (a) TEM image of confined CsPbIBr_2_ perovskite within ZrSBA-15; (b) TEM image of CsPbIBr_2_ perovskite crystallized on the surface of ZrSBA-15; (c) SAED of confined CsPbIBr_2_ perovskite within ZrSBA-15; (d) SAED of confined CsPbIBr_2_ perovskite crystallized on the surface of ZrSBA-15 in (b).


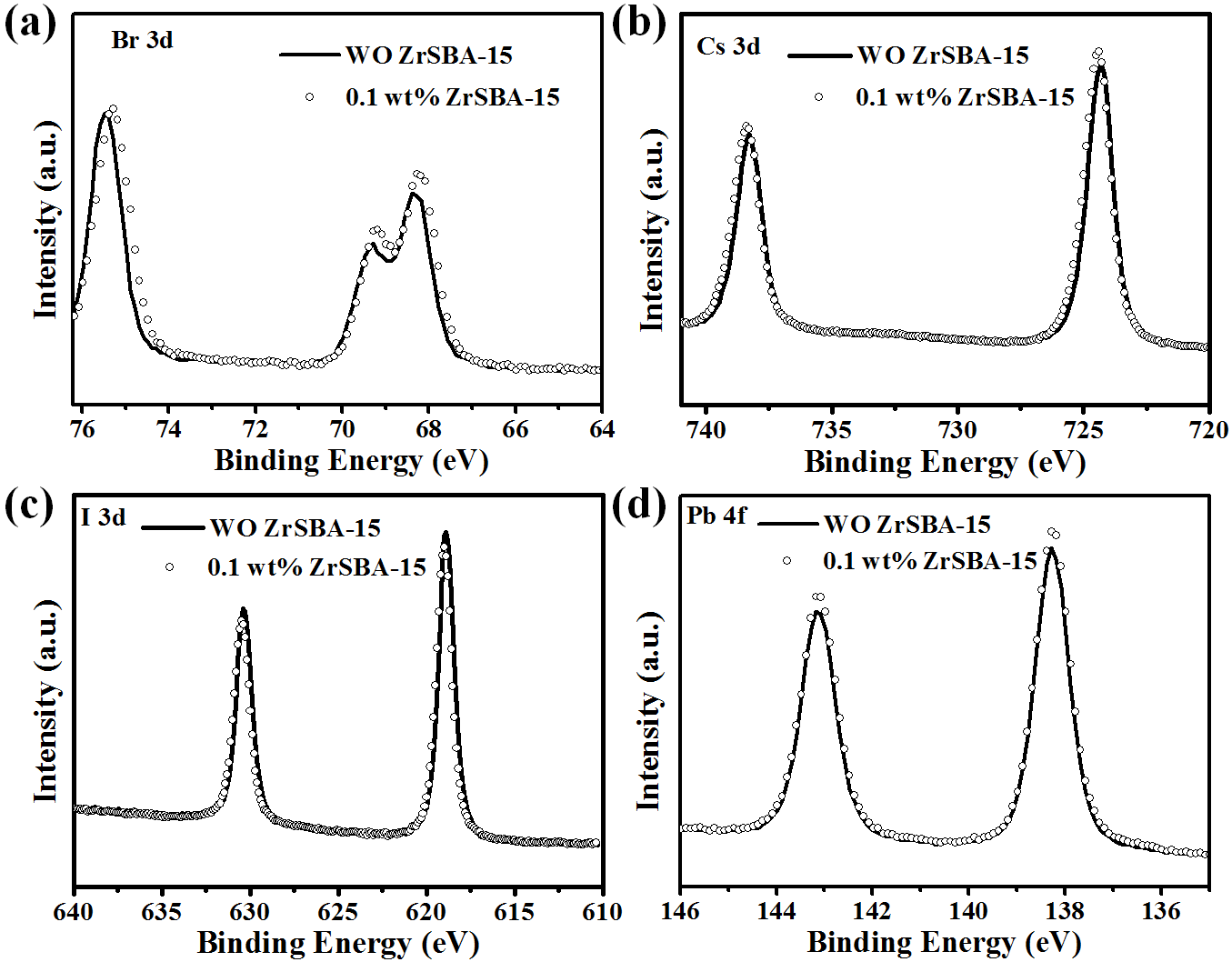


Figure S4. High resolution XPS spectra of CsPbIBr_2_ perovskite films without ZrSBA-15 and with 0.1 wt% ZrSBA-15; (a) Br 3d; (b) Cs 3d; (c) I 3d; (d) Pb 4f.


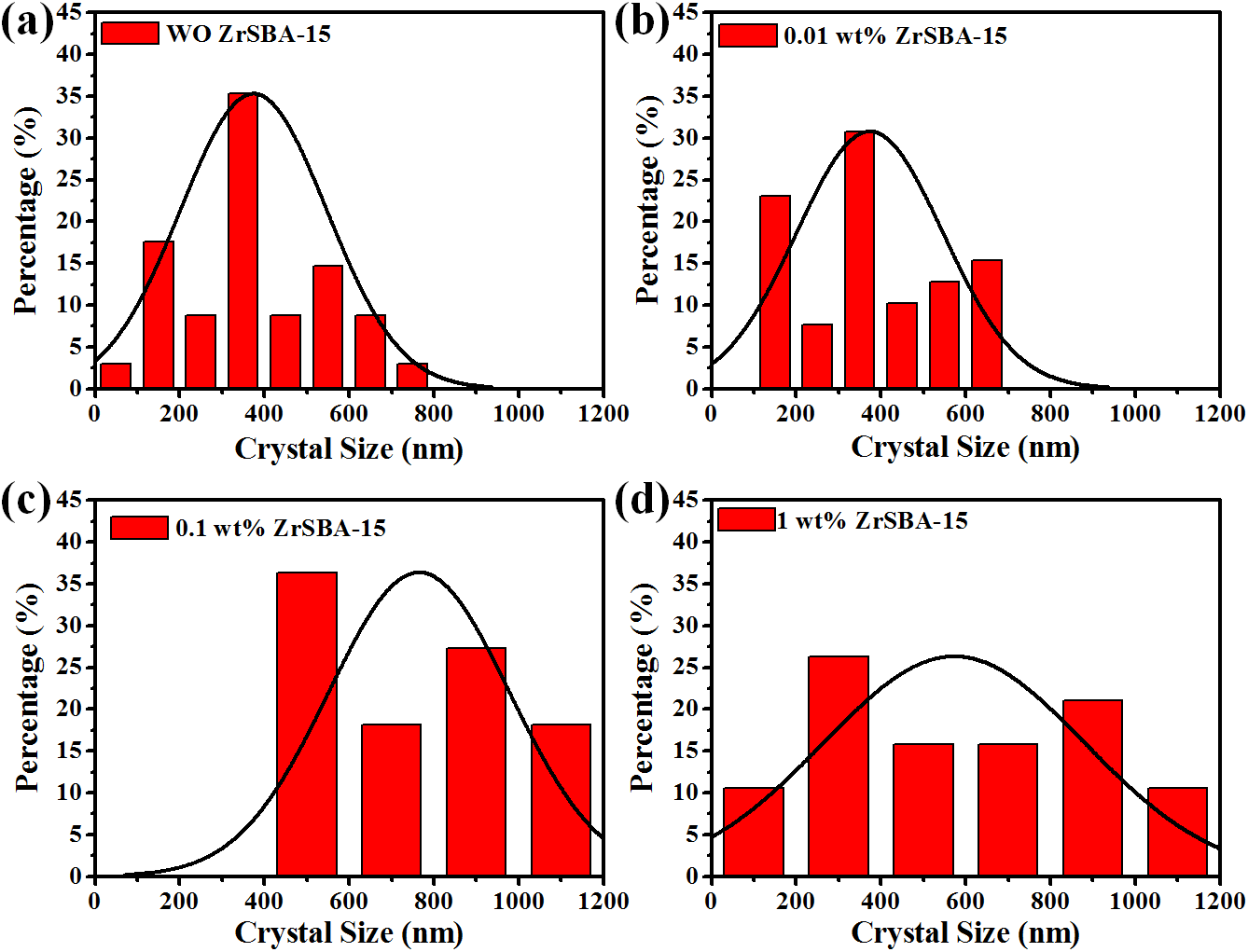


Figure S5. Crystal size distributions of various CsPbIBr_2_ perovskite films; (a) without ZrSBA-15; (b) with 0.01 wt% ZrSBA-15; (c) with 0.1 wt% ZrSBA-15; (d) with 1 wt% ZrSBA-15.


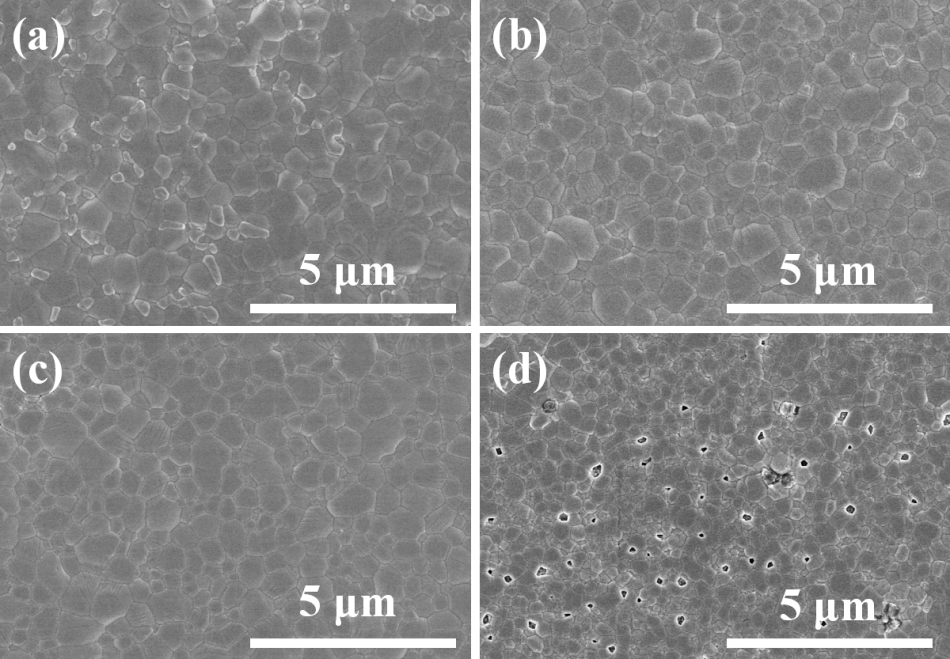


Figure S6. Top view SEM images of various CsPbIBr_2_ perovskite films; (a) without ZrSBA-15; (b) with 0.01 wt% ZrSBA-15; (c) with 0.1 wt% ZrSBA-15; (d) with 1 wt% ZrSBA-15.


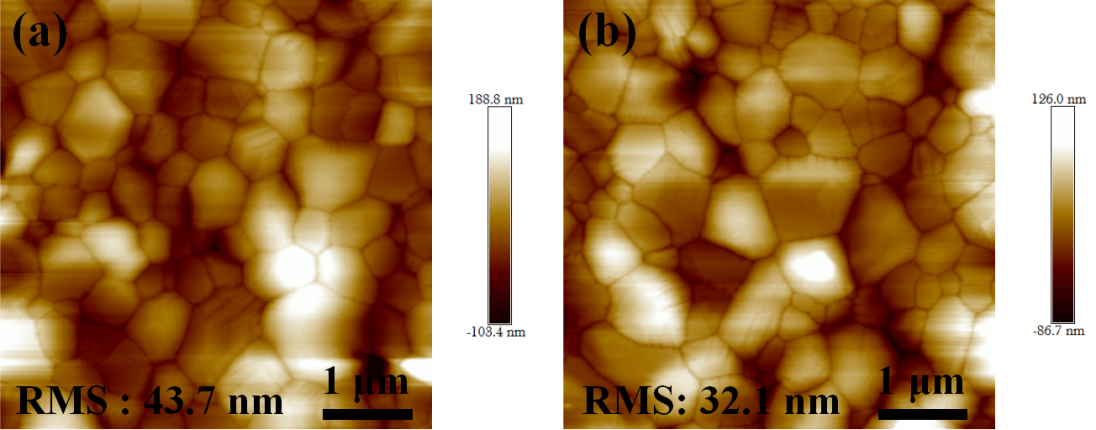


Figure S7. AFM images of CsPbIBr_2_ perovskite films; (a) without ZrSBA-15; (b) with 0.1 wt% ZrSBA-15.


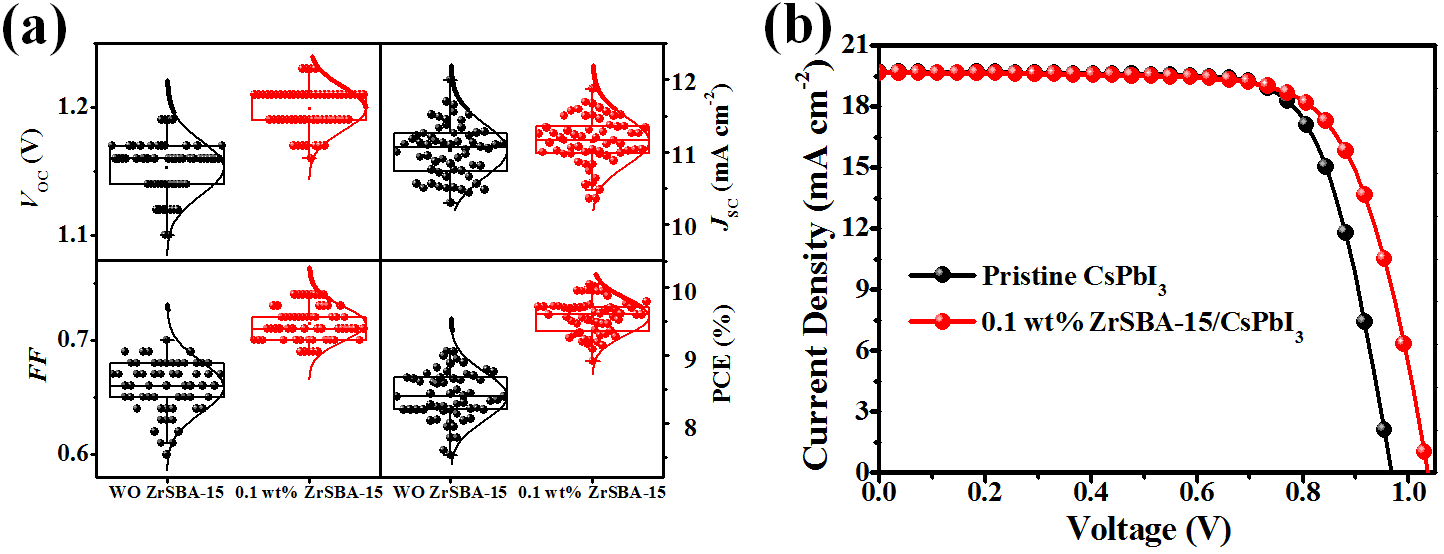


Figure S8. (a) Photovoltaic parameters distributions of CsPbIBr_2_ PSCs without and with 0.1 wt% ZrSBA-15; (b) *J*-*V* curves of CsPbI_3_ PSCs without and with 0.1 wt% ZrSBA-15.


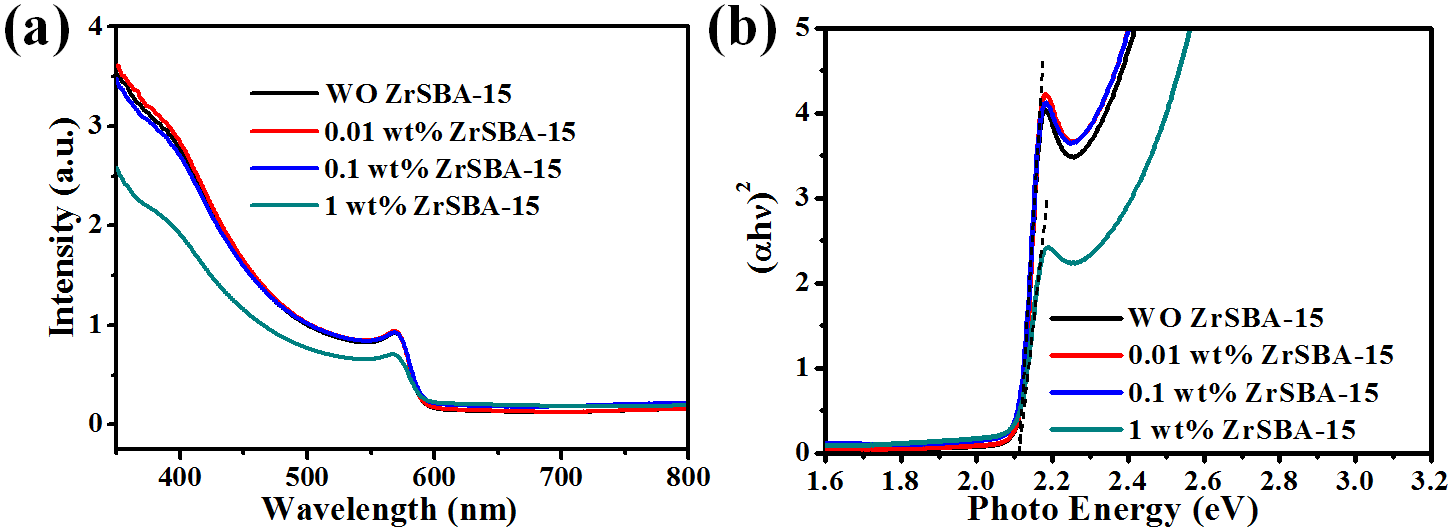


Figure S9. (a) UV-vis adsorption spectra of various CsPbIBr_2_ perovskite films without and with ZrSBA-15; (b) Corresponding bandgap plots of (a).


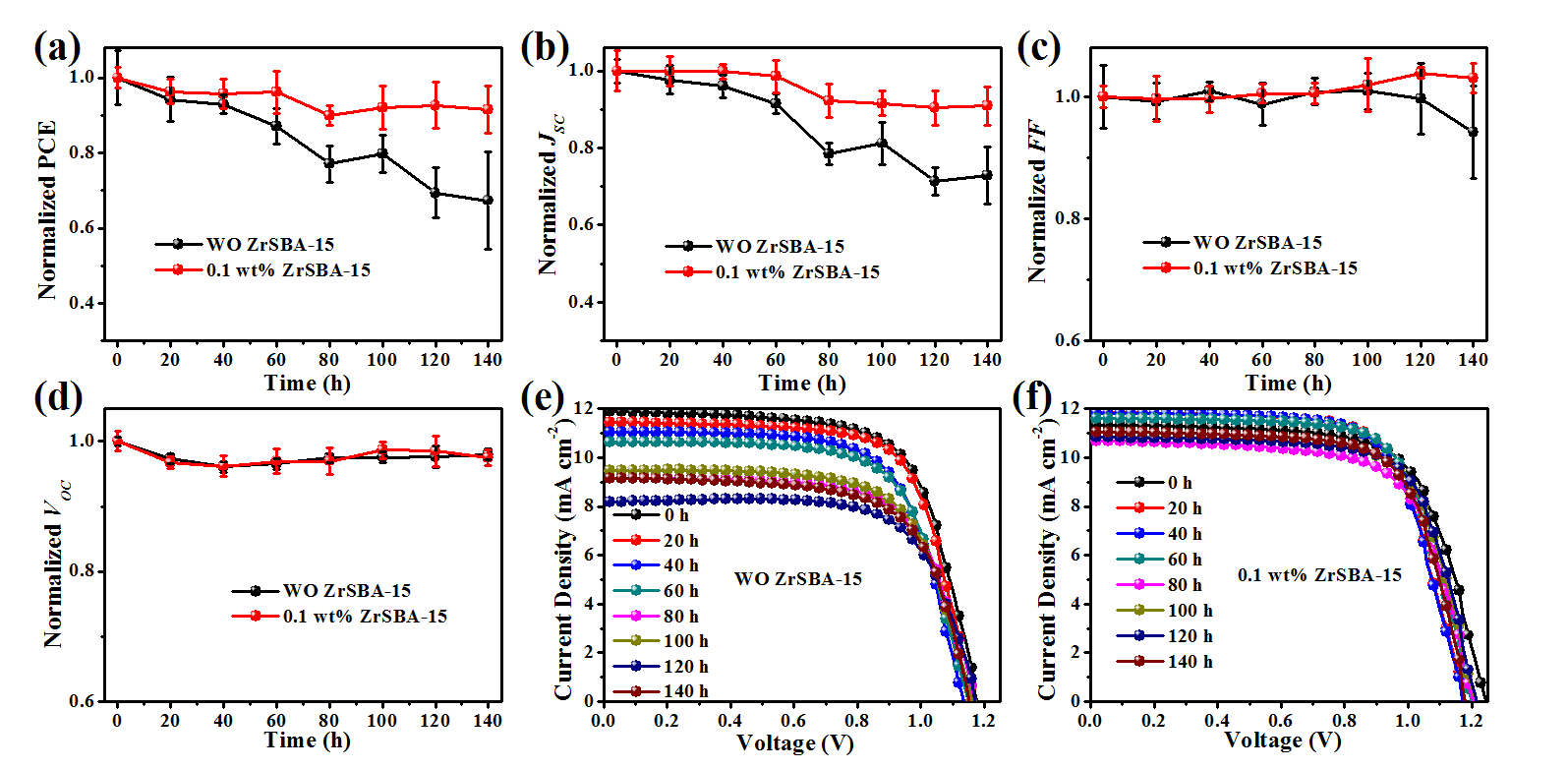


Figure S10. Stability parameters of CsPbIBr_2_ PSCs without and with 0.1 wt% ZrSBA-15 (a) Normalized PCE; (b) Normalized *J_SC_*; (C) Normalized *FF*; (d) Normalized *V_OC_*; (e), (f) Corresponding *J*-*V* curves in stability test.


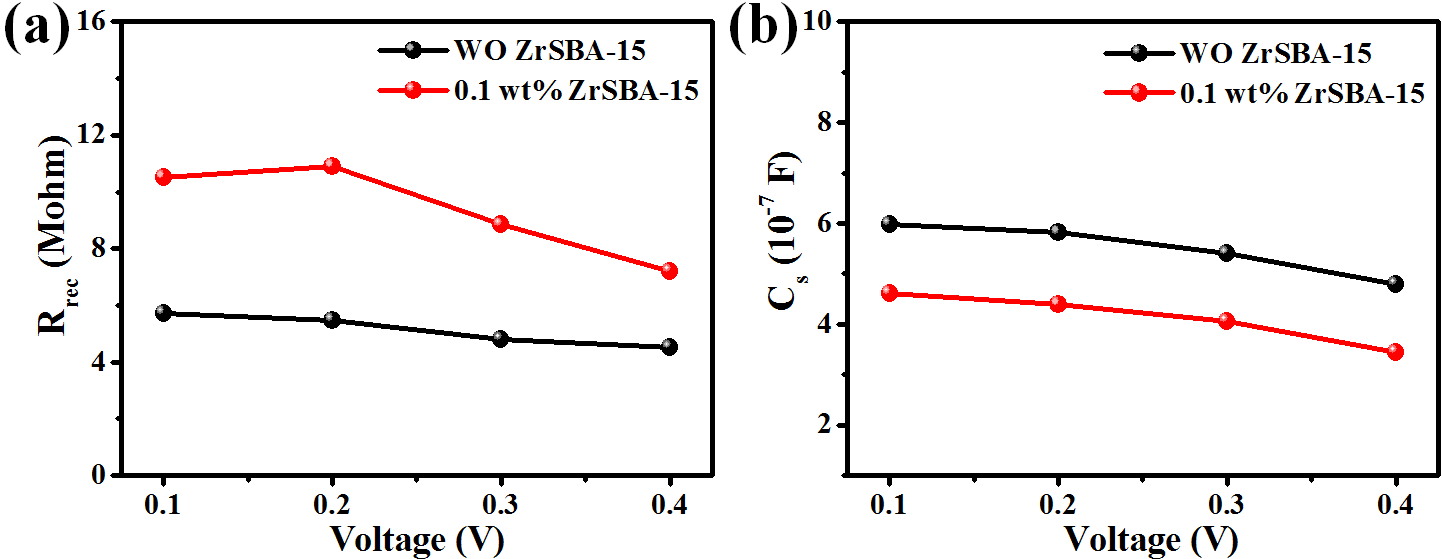


Figure S11. (a) *R*_rec_ as a function of bias voltages; (b) *C*_s_ as a function of bias voltages.

Table S1. Photovoltaic parameters of CsPbIBr_2_ PSCs without and with 0.1 wt% ZrSBA-15.

|  | | *V_OC_* (v) | *J_SC_* (mA cm^-2^) | *FF* | PCE (%) |
| --- | --- | --- | --- | --- | --- |
| WO ZrSBA-15 | Average | 1.14 ± 0.03 | 10.85 ± 0.37 | 0.65 ± 0.02 | 8.29 ± 0.37 |
|  | Champion | 1.16 | 11.23 | 0.66 | 8.67 |
| 0.1 wt% ZrSBA-15 | Average | 1.20 ± 0.02 | 11.15 ± 0.33 | 0.71 ± 0.01 | 9.54 ± 0.28 |
|  | Champion | 1.23 | 11.35 | 0.72 | 10.04 |
